# Germinated Brown Rice: A Natural Source of Bioactive Compounds Boosting Human Health

**DOI:** 10.1155/ijfo/8231866

**Published:** 2026-08-05

**Authors:** Georgiana Bosco, Valentina Taverniti, Alessandra Pino, Cinzia Caggia, Katarzyna Stadnicka, Cinzia L. Randazzo

**Affiliations:** ^1^ Department of Agriculture, Food and Environment (Di3A), Università di Catania, Catania, Italy, unict.it; ^2^ Sacco S.R.L., Cadorago, Italy; ^3^ ProBioEtna srl, Spin-off of University of Catania, Catania, Italy; ^4^ Faculty of Health Sciences, Collegium Medicum, Nicolaus Copernicus University, Bydgoszcz, Poland, umk.pl

**Keywords:** bioactive compounds, functional foods, germination, health benefits, nutraceutical applications

## Abstract

Germinated brown rice (GBR) has gained considerable attention as a functional food due to both nutritional and bioactive profiles as well as health‐promoting properties. The present narrative review is aimed at summarizing and discussing available research on GBR, focusing on bioactive composition and potential to support human well‐being. Based on available data, the germination process of brown rice enhances the bioavailability of key bioactive compounds, including polyphenols, *γ*‐aminobutyric acid, *γ*‐oryzanol, vitamins, and dietary fibers. Additionally, a substantial body of evidence supports the ability of GBR to exert beneficial physiological effects on the host, such as modulation of lipid and glucose metabolism, antioxidant and anti‐inflammatory activities, and positive influences on gut microbiota composition. The use of GBR in formulating functional foods and nutraceutical supplements further highlights its versatility as a promising strategy in supporting human well‐being.

## 1. Introduction

Rice (*Oryza sativa* L.) is one of the most widely consumed cereals worldwide, and based on postharvest processing, it is mainly classified into brown rice (BR) and white rice (WR). Depending on rice genotype, growing environment, and geographical origin, BR is generally characterized by higher concentrations of dietary fibers (DFs) and bioactive compounds compared to WR, as the bran and embryo are preserved during milling. These include functional lipids, amino acids, vitamins, phenolic compounds, *γ*‐aminobutyric acid (GABA), phytosterols, and minerals [1].

Germinated brown rice (GBR) is produced by soaking the BR grain in water and has been investigated as a potential functional food due to the enhanced nutritional profile. Although depending on genotype, germination conditions, and analytical methodology, the germination of BR grains has been associated with increasing content of bioactive compounds, such as polyphenols, vitamins, inositol, GABA, and *γ*‐oryzanol, whereas for minerals, the primary reported effect is an improvement in bioavailability [2]. GBR also contains fermentable DF, suitable as a substrate for the growth of beneficial gut microorganisms. Accumulating evidence, mainly derived from in vitro studies and animal models, suggests that GBR may be associated with various physiological benefits, including modulation of lipid metabolism, blood pressure regulation, glycemic control, and reduced risk of cardiovascular and metabolic disorders as well as potential antiproliferative effects [2, 3]. GBR has also been used to produce several value‐added foods, including beverages, bread, noodles, fermented products, and probiotic‐enriched foods [4–9].

The aim of the present narrative review is to summarize and critically discuss recent advances in research on GBR, with a focus on bioactive composition, potential health benefits, and current applications, while acknowledging the strengths and the limitations of the available scientific evidence.

## 2. Review Structure

This article reviewed the scientific literature on GBR, with a particular focus on nutritional composition, profile of bioactive compounds, and potential health benefits, as well as possible applications for the formulation of functional foods and dietary supplements. A comprehensive search of the electronic databases PubMed, Scopus, and Web of Science was conducted, and scientific research published from 2015 to 2025 relevant to the scope of the review was selected. Search terms included “brown rice,” “germinated brown rice,” “sprouted brown rice,” and “pre‐germinated brown rice” in combination with keywords such as “germination,” “bioactive compounds,” “nutritional composition,” “dietary fiber,” “GABA,” “gamma‐oryzanol,” “gut microbiota,” “functional foods,” and “health benefits.”

## 3. GBR

**Figure 1:**
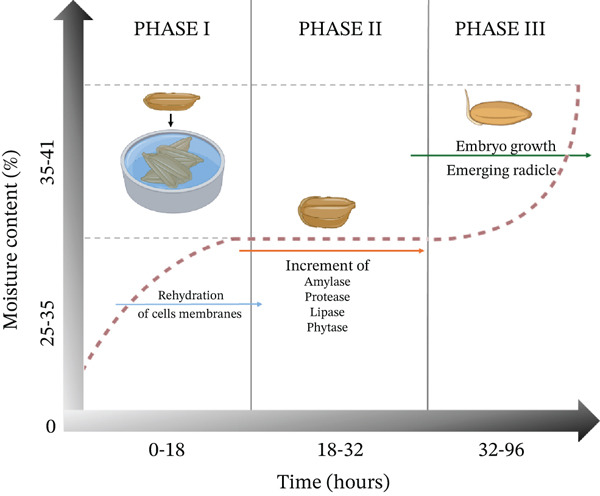
Germination process of brown rice grains in water.

GBR, also known as sprouted BR, derives from the soaking and sprouting of BR grains in water, a process that significantly enhances the bioavailability of nutrients by neutralizing phytic acid (PA) [2]. GBR is obtained through a biological process initiated by water absorption. As schematized in Figure 1, although the duration of each phase varies depending on rice genotype, water temperature, and soaking conditions, the germination process could be divided into three main phases: (i) imbibition, (ii) activation/lag phase, and (iii) germination [10]. During the imbibition phase, which typically occurs within the first 0–18 h of germination [11], the grain rapidly absorbs water, causing the embryo to swell and brighten. Usually, after 18–32 h during the activation/lag phase, endogenous enzymes, responsible for the metabolic processes, are activated. Among these, the *α*‐amylase plays a key role in breaking starch, while *β*‐amylase further degrades it, producing maltose [12]. Other enzymes involved include protease, which hydrolyses storage proteins into amino acids; RNase, which degrades RNA, supporting nucleic acid turnover; phytase, which releases phosphate from phytate, improving mineral availability; and glutamate decarboxylase (GAD), which converts glutamate to GABA [10]. These enzymes primarily facilitate the mobilization of stored nutrients and the synthesis of bioactive compounds. As germination progresses (from 32 to 96 h), the coleoptile begins to emerge from the embryo. The seedling length and moisture content increase steadily during this stage; the radicle breaks the seed coat and starts to emerge, marking the visible completion of germination [1, 13]. Due to the activation of hydrolytic enzymes, germination induces significant changes in biochemical, nutritional, and sensory characteristics, enhancing the bioavailability of nutrients and making the GBR a functional food with a valuable content of health‐promoting molecules [14]. Table 1 summarizes nutrients and bioactive compounds affected by germination. In addition, quantitative changes between BR and GBR, as well as health‐promoting features, are reported. Carbohydrates are substantially affected by germination since the hydrolytic enzymes are the first to be activated in response to germination conditions. Among these, *α*‐amylase is responsible for the fragmentation of starch stored in grains, converting it to maltose. At the same time, pullulanase is responsible for the production of glucose monomers used in cellular respiration and energy production [15]. According to that, germination is an effective process that increases the digestibility of both fast‐digesting and slow‐digesting starch, suggesting a loosening of the starch–protein and starch–lipid complexes [16]. Concerning proteins, during the first stage of germination, free amino acids may increase due to the hydrolytic activity of proteases, which break down storage proteins into smaller peptides and free amino acids. The observed accumulation largely reflects protein mobilization rather than de novo synthesis [17]. The amount of certain essential amino acids, such as leucine, lysine, and tryptophan, mainly increases during the early stages of germination [18]. For some substances, such as PA, the germination of rice grains is effective in reducing their presence. Germination also acts on the lipidic fraction, as reserved lipids are degraded by the action of lipolytic enzymes and oxidation reactions to generate energy by *β*‐oxidation and the glyoxylate pathway [19]. By comparing the profile of metabolites in rice grains (white, black, and red) before and after germination, an increase in fatty acid content, mainly *α*‐linolenic acid, was revealed [20]. This increase has been attributed to the hydrolytic activity of endogenous lipases activated during germination, which degrade stored lipids, releasing free fatty acids [2]. Additionally, among bioactive compounds, GABA shows the most consistent and well‐documented increase following germination, primarily due to the activation of GAD, which catalyzes the decarboxylation of L‐glutamate to GABA [10]. GAD activity is promoted by intracellular acidification and by increased Ca^2+^/calmodulin signaling during germination [10]. Changes in other compounds, including tocopherols, tocotrienols, and *γ*‐oryzanol, have also been reported [1, 21, 22].

**Table 1:** Macro‐ and micronutrients, as well as bioactive compounds in brown rice (BR) before and after germination (GBR), along with associated key functions in health maintenance.

Nutrient	Compound	BR	GBR	Key function and health benefit	References
Total carbohydrates (g/100 g)		72–76	64–72	Main energy source	[23–27]
Protein (g/100 g)		8.2–11.1	9.9–15	Essential for tissue structure, muscle maintenance, and immune function	[27–30]
Fat (g/100 g)		2.8	2.6	Heart, brain, and immune health support	[30–32]
Dietary fiber (g/100 g)		2.5–8.3	1.3–9.3	Modulation of gut microbiota, reduces risk of obesity, diabetes, and cardiovascular disease	[27, 30, 33, 34]

Amino acids, branched‐chain amino acids (BCAAs), and derivatives (mg/100 g)	GABA	0.6–7.1	2.1–253.4	Neuroprotection, antistress, and improved metabolism	[1, 9, 35–37]
	Leucine	103	114	Reduced muscle soreness, maintenance of muscle mass, and improved recovery	[38–41]
	Isoleucine	48	54		
	Valine	67	75		

Vitamins (mg/100 g)	Vitamin E	1.38	1.89–2	Antioxidant and metabolic support	[42–44]
	Vitamin B (B2, B6, and B9)	0.68–1.66	0.33–3.63		

Minerals (mg/100 g)	Calcium	106	100–290	Essential for various physiological functions	[43, 45, 46]
	Iron	4.65	2.02–9.43		
	Zinc	1.70	1.74–2.23		
	Selenium	82.20 (*μ*g/100 g)	58.6–90 (*μ*g/100 g)		

Total phenolic compounds (GAE)/g	Ferulic acid, coumaric acid, cinnamic acid, syringic acid, caffeic acid, sinapinic acid, tricin, and anthocyanins	1.0–1.01	1.31–19.5	Antioxidant, anti‐inflammatory, and anticancer properties	[47–51]

*γ*‐Oryzanol (mg/100 g)	*γ*‐Oryzanol	34.7	52.73	Cholesterol‐lowering property and antioxidant activity	[8, 52–54]

Other secondary metabolites (mg/100 g)	Momilactones (A and B)	0–2	1.89–4.10	Antimicrobial and anti‐inflammatory activities	[55–57]
	Phytic acid	820–2620	230–1000	Antinutrient	[58, 59]

## 4. Potential Health Benefits of the Main GBR Compounds

### 4.1. DF

DF exerts well‐documented health‐promoting effects. In fact, DF cannot be digested and absorbed in the small intestine [60]. However, microorganisms present in the gut microbiota can ferment DF, promoting the growth of beneficial bacteria and the production of metabolites with positive health effects [61]. More broadly, DF intake has been associated with several health benefits, including reduced cardiovascular risk [62], lower incidence of obesity and Type II diabetes [63], and reduction of the glycemic index after meals [64].

The germination of BR leads to changes in both the total amount and the composition of DF, including both the soluble dietary fiber (SDF) and insoluble dietary fiber (IDF) fractions, although the magnitude of these changes varies depending on rice variety and germination conditions [43]. Specifically, *α*‐amylase activation leads to starch hydrolysis, releasing simple sugars that are redirected toward the biosynthesis of new structural cell wall polymers, including cellulose, hemicellulose, and lignin, directly increasing the DF content [7, 65]. Additionally, starch depletion determines a relative increase in the proportion of nondigestible carbohydrates in the grain dry matter [2].

The potential prebiotic role of GBR‐derived DF has been investigated in a limited number of studies. Ren et al. [66] evaluated the hypolipidemic potential of GBR and germinated black‐pigmented BR in a rat model. The authors revealed that the dietary intervention contributed to the remission of hyperlipidemia by alleviating the dysbiosis of gut microbiota. In particular, a decrease in the *Firmicutes*/*Bacteroidetes* ratio was observed. At the genus level, some differential microbial genera relating to lipid metabolism were also determined, such as *Lachnospira*, *Ruminococcus*, *Phascolarctobacterium*, *Dorea*, *Turicibacter*, and *Escherichia–Shigella.* The same author conducted a 3‐month dietary intervention on patients with hyperlipidemia, receiving GBR and polished rice. Analyses of the gut microbiota at the genus level revealed that GBR supplementation determined the increase of *Bacteroides*, *Faecalibacterium*, *Dialister*, *Prevotella*, and *Bifidobacterium*, while *Escherichia–Shigella*, *Blautia*, *Romboutsia*, and *Turicibacter* were decreased [3]. Similarly, Zhao et al. [67] investigated, in high‐fat diet‐fed mice, the effects of BR and GBR supplementation on fecal short‐chain fatty acids (SCFAs), gut microbiota, metabolism, and inflammation. Results revealed a distinct profile of both SCFAs and gut microbiota. Based on these findings, the increases in SCFAs in the feces of treated mice were associated with improvements in gut microbiome and metabolic and inflammatory profiles, contributing to antidiabetic and anti‐inflammatory effects.

### 4.2. Amino Acids and Derivatives

Compared to non‐GBR, the GBR is characterized by increased levels of amino acids, including lysine, methionine, leucine, isoleucine, threonine, phenylalanine, and valine. Notably, the intake of amino acids is positively correlated with various health benefits, including improvement in metabolic health, maintenance of muscle mass, and a potential role in disease prevention. In particular, branched‐chain amino acids (BCAAs), including valine, leucine, and isoleucine, were reported to directly stimulate the release of insulin. Accordingly, Wan et al. [41] conducted a randomized controlled trial with the aim of addressing the relationship between GBR and BCAA metabolism in Type II diabetes mellitus patients. Results revealed that the consumption of GBR (100 g/day for 3 months) improved fasting blood glucose and insulin resistance. Although requiring further validation, one of the possible mechanisms proposed by the authors involves an increased mRNA expression of two catabolic enzymes related to BCAA catabolism, namely, branched‐chain aminotransferase and branched‐chain keto acid dehydrogenase, which could accelerate BCAA degradation.

Among amino acids, GBR is rich in GABA, a four‐carbon nonprotein amino acid acting as an inhibitory neurotransmitter in the mammalian cortex, whose production and biological functions have been extensively investigated [1]. The effect of GBR on vascular cognitive impairment and glutamate‐induced toxicity was evaluated and compared with that of pure GABA in mice with mild cerebral hypoperfusion [36]. By using a mouse model of modified bilateral common carotid artery occlusion (BCCAo) and glutamate‐induced HT22 cell death, the authors highlighted that the treatment with GBR protected against cell damage in the brain and attenuated cerebral ischemia–induced learning and memory deficits. Moreover, a more pronounced protective capability against glutamate‐mediated neuronal toxicity in HT22 murine hippocampal neuronal cells was associated with GBR than pure GABA [36]. The protective effects of GBR via suppression of glutamate‐induced oxidative stress in hippocampal neuronal HT22 cells may be a promising strategy for use as a nutraceutical for neurodegenerative diseases, both therapeutically and prophylactically. According to that, Promtang et al. [37] demonstrated that GBR (100 *μ*g/mL) can exert neuroprotective and antiapoptotic properties against glutamate toxicity, induced in differentiated HT22 cells, through the GABA_A_ receptor. Interestingly, the GBR exhibited a better neuroprotective effect than a pure commercial GABA compound (0.115 *μ*M). Beyond neuroprotection, GBR is also able to exert systemic antioxidant effects, as evidenced by studies showing reductions in oxidative stress markers and improvements in inflammatory profiles in both in vitro and animal models [58].

### 4.3. Vitamins

Vitamin E, especially *α*‐tocopherol, *α*‐tocotrienol, and *β*‐tocopherol, exerts antioxidant, antiatherosclerosis, and hepatoprotective effects [42]. It has been demonstrated that, at the cellular level, *α*‐tocopherol reduces lipid hydroperoxide formation and stabilizes polyunsaturated fatty acids (PUFAs) in biological membranes, thus preserving membrane integrity [68]. Moreover, a randomized clinical trial demonstrated that *α*‐tocopherol supplementation significantly reduced serum TNF‐*α* and C‐reactive protein in adults with metabolic syndrome, suggesting that supplementation with vitamin E can attenuate markers of systemic inflammation [69]. Furthermore, studies have shown that vitamin E may improve serum lipid profile by reducing oxidative stress and inflammation [70].

Notably, GBR exhibits a richer vitamin profile compared to non‐GBR or WR [71]. It has been demonstrated that, in GBR, changes in vitamin E content are correlated with rice variety and germination time. A study investigating four BR cultivars (Guanghei1, Guichao2, Guanghong6, and Xiangyaxiangzhan) revealed that the total vitamin E content increased during the germination process in all cultivars. This increase was attributed to the upregulation of MPBQ/MT2, *γ*‐TMT, and TC genes, which are responsible for the enhanced accumulation of *α*‐tocopherol, *α*‐tocotrienol, and *β*‐tocopherol [44]. However, *γ*‐tocopherol content decreased significantly across all cultivars, and several isomers, including *δ*‐tocopherol/tocotrienol and *β*‐tocotrienol, were below the detectable limit [44]. Among vitamins, GBR shows a significant content in vitamin B, particularly vitamin B2 (riboflavin), which exerts antioxidant activity, stimulates the immune system, and prevents some chronic diseases [72]. In a study by Ukpong et al. [43], the content of vitamin B in BR, milled rice (MR), and GBR was evaluated. The researchers observed that the levels of vitamins B1, B2, B3, and B6 in MR were significantly lower than those in BR. Furthermore, vitamins B2 and B3 were significantly higher in GBR, while vitamins B1 and B6 showed no significant differences between BR and GBR, indicating that germination had no significant impact on these two specific compounds.

### 4.4. Minerals

Minerals play a crucial role in general health, contributing to tissue maintenance and bone and tooth health, regulating and coordinating body functions, and acting as cofactors for various enzyme systems [45]. The effect of soaking and germination on the mineral content of BR has been investigated in several studies, although the available health‐related evidence is limited. As highlighted in a recent comprehensive review, germination does not result in meaningful changes in mineral levels in BR [58]. Differently, Omohimi et al. [49], evaluating the effect of germination on nutritional composition and technofunctional qualities of three BR varieties (*Ofada*, FARO 44, and Igbemo), observed the enhancement of all minerals analyzed. Germination can also affect the content of potentially toxic microelements. In this context, a reduction of lithium, aluminum, arsenic, cadmium, lead, and mercury was observed in Longjing 21 BR throughout germination. Additionally, changes in the content of essential microelements (e.g., boron, manganese, iron, cobalt, copper, zinc, and selenium) were associated with germination [46]. The reduction of toxic elements may be partially explained by the release of water‐soluble metals into the soaking medium and their diffusion during repeated washing and water replacement. Although soaking, washing, and cooking can significantly reduce the concentration of potentially toxic microelements (e.g., arsenic, cadmium, and lead), the extent of removal is influenced by several parameters such as soaking time, temperature, water composition, and processing conditions. Furthermore, germination‐induced metabolic and structural changes within the grain may alter metal distribution and extractability, although direct evidence for these mechanisms in GBR is still limited [73–75].

### 4.5. Phenolic Compounds

Phenolic compounds are a diverse group of phytochemicals naturally found in plant‐based foods. They play a key role in human health due to their antioxidant, anti‐inflammatory, and antimicrobial properties [48]. Bound phenols can have beneficial effects throughout the digestive tract and help protect cells from DNA damage and lipid peroxidation [50]. Phenolic acids are extensively reported across many cereals, although their content can vary considerably among cultivars. The rice bran contains high amounts of phenolic compounds, and the germination of BR induces a significant increase in the content of free phenolic acids and insoluble‐bound phenolic compounds [58]. This increase has been mainly attributed to the activation of hydrolytic enzymes during germination, such as esterases, cellulases, and xylanases, which promote cell wall degradation and the release of phenolic acids previously bound to structural polysaccharides. In addition, germination may stimulate the phenylpropanoid pathway, leading to de novo synthesis of phenolic compounds, while structural modifications of the cell wall can increase the extractability of both free and bound phenolic fractions [76–79]. Particularly, ferulic acid, p‐coumaric acid, and caffeic acid are among the most extensively investigated phenolic acids because of their potential health‐promoting properties in preventing the risk of liver cirrhosis and cancer. Specifically, simple phenols (i.e., ferulic acid and caffeic acid) extracted from light BR were associated with potential chemopreventive effects [79]. Additionally, in animal feeding studies, supplementation with the cell wall–bound fraction of rice bran resulted in the reduction of hypertension, hyperlipidemia, and hyperglycemia [58].

### 4.6. *γ*‐Oryzanol

*γ*‐Oryzanol is a complex mixture of ferulic acid esters of phytosterols and triterpene alcohols predominantly found in rice bran. Recently, *γ*‐oryzanol has gained increasing attention for its biological activities, including antioxidant, anti‐inflammatory, and lipid‐lowering effects, as well as the ability to modulate cellular metabolic pathways in both in vitro and in vivo models [52]. *γ*‐Oryzanol is abundant in the bran layer of BR, and although both composition and concentration vary significantly among rice varieties and are influenced by genetic, environmental, and technological factors, its content is preserved or increased in GBR [58]. Clinical observation in obese, prediabetic men revealed that daily intake of BR was associated with attenuation of bodyweight gain, improvement in impaired glucose tolerance and insulin resistance, and reduced preference for fatty foods [80]. Additionally, a study conducted on mice showed that *γ*‐oryzanol in BR can cross the blood–brain barrier, reducing the preference for high‐fat diets, suggesting that *γ*‐oryzanol may contribute to ameliorating metabolic dysregulation associated with feeding behavior [80]. Similarly, pre‐GBR containing *γ*‐oryzanol, GABA, flavonoids, and anthocyanidin inhibits high‐fat diet‐induced metabolic syndrome in mice by reducing body and liver weight gain and improving glucose tolerance and insulin sensitivity [81]. As recently reported, *γ*‐oryzanol might play a crucial role in managing menopause‐related symptoms and complications. The administration of *γ*‐oryzanol for 20 days in ovariectomized mice (a model of menopause) attenuated depressive‐like behavior through upregulation of ER*β*‐mediated hippocampal neuronal nitric oxide synthase expression and activation of ERK‐CREB‐BDNF signaling pathways. These findings suggest that *γ*‐oryzanol may mimic some of the neuroprotective actions of estrogen and contribute to attenuating menopausal depression [82].

### 4.7. Other Secondary Metabolites

Among the secondary metabolites, momilactones can be naturally found in rice (*Oryza* lineage) and enhanced through germination. As reported in the *Koshihikari* variety, increasing salinity and prolonging germination for 4 days led to an increase in both Momilactone A (MA) and Momilactone B (MB) [56]. Momilactones are bioactive diterpenoids, and although primarily known for their allelopathic properties, MA and MB have been associated with health‐promoting properties [54]. In this context, Quan et al. [83], by in vitro testing both MA and MB from rice bran, demonstrated inhibitory activities on pancreatic *α*‐amylase and *α*‐glucosidase, which were significantly higher than *γ*‐oryzanol, a well‐known diabetes inhibitor. Another class of secondary metabolites of interest in GBR is PA. PA can bind essential dietary minerals (e.g., iron, zinc, calcium, and magnesium), forming insoluble complexes which are poorly absorbed in the human gastrointestinal tract [84]. Additionally, PA can form stable complexes with proteins, which may result in decreased protein solubility, enzymatic activity, and proteolytic digestibility [58]. Through the germination process, phytase enzymes are activated, which decompose PA, releasing *myo*‐inositol and inorganic phosphate, improving the bioavailability of minerals and the nutritional profile of GBR. In particular, *myo*‐inositol has been reported to exert several beneficial effects on humans, such as the modulation of serotonin pathways, reducing symptoms of depression and panic disorder [85–87], and its supplementation can be used in subfertile women with and without polycystic ovary syndrome (PCOS) [88]. However, evidence of beneficial effects of *myo*‐inositol derived from GBR is currently lacking and warrants further investigation.

## 5. Use of GBR for the Formulation of Functional Foods

As summarized in Table 2, GBR has been evaluated as a suitable ingredient for the formulation of functional foods, such as beverages, bread, noodles, fermented foods, and snacks. In this context, Jabeen et al. [8] developed a novel GABA‐rich functional beverage using GBR from the Jhelum rice variety with the addition of powdered sugar, chocolate powder, sodium carboxymethyl cellulose, dairy whitener, and essence. Data on the total GABA content revealed a concentration of GABA (42.12 mg/100 g) corresponding to 14% of the recommended daily intake (300 mg/day) according to FAO/WHO guidelines, highlighting the potential contribution of a GABA‐enriched beverage as a functional food. The suitability of GBR to produce probiotic fermented products was investigated by Pino et al. [6]. Four different fermented products were formulated, and microbiological and chemical profiles, presence of bioactive compounds, antioxidant activity, sensory attributes, and shelf life during refrigerated storage were monitored. The authors highlighted that the fermentation process increased the GABA content (359 *μ*g/mL) and oryzanol (96.3 *μ*g/mL) compared to the unfermented beverage (178.7 and 44.80 *μ*g/mL, respectively) [6]. Beyond beverages, GBR has also been applied in the formulation of value‐added cereal products. In the study conducted by Chen et al. [7], the impact of BR germination on cooking, textural, sensory, and nutritional properties of BR noodles was investigated in comparison to ungerminated BR noodles. Overall, data showed that germination improved cooking and textural properties as well as sensory quality. In particular, the content of GABA, free phenolic acid, and bound phenolic acid increased by 53.43%, 21.71%, and 7.14%, respectively, while the content of resistant starch decreased by 21.55% compared to ungerminated BR noodles. This evidence suggests that sprouting is a promising strategy for improving the edible quality and nutritional properties of BR noodles [7]. Recently, a sourdough bread formulated with *Latilactobacillus sakei* and GBR was developed and characterized. Compared to the control bread, the use of sourdough containing *L. sakei* resulted in a bread with higher content of phenolic compounds and in vitro antioxidant activity, demonstrating the technological suitability of integrating GBR with lactic acid bacteria in bread making [9]. The versatility of GBR extends further, as recent studies have evaluated the nutritional profile of WR‐ and GBR‐based snacks, revealing a higher content of rapidly digestible starch and a lower content of slowly digestible starch in the two products, respectively [89]. Moreover, the feasibility of producing ice cream using GBR flour (KDML105 variety) as a prebiotic source for the probiotic strain *Latilactobacillus acidophilus* LA‐5 was also examined. Four different concentrations of GBR and corn flour were used at different ratios. Although physicochemical and microbiological analyses showed that all formulations met legislative requirements and exhibited good sensory acceptability, the formulation with equal percentages of GBR flour and corn flour showed greater viability of the probiotic strain after 30 days of storage at controlled temperatures [4]. Collectively, these studies indicate that GBR represents a versatile and technologically suitable ingredient for the development of value‐added food products. However, clinical or intervention studies directly demonstrating health effects following consumption of these specific products are currently lacking.

**Table 2:** Summary of studies on the use of germinated brown rice (GBR) in functional foods, dietary supplements, and other applications.

		Aim	Main outcomes	Reference
Functional foods	GABA‐enriched GBR beverage	Develop a GBR‐based functional beverage enriched in GABA	↑ *γ*‐Oryzanol	[8]
			↑ Phenolic compounds	
			↑ Niacin	
			↑ GABA	
			↑ Antioxidant activity	
			↑ Good sensory acceptability	
	Probiotic‐fermented GBR products	Evaluate GBR suitability for fermented probiotic foods	↑ *γ*‐Oryzanol	[6]
			↓ Phytic acid; probiotic survival > 9 log CFU during storage, good shelf‐life stability	
	GBR noodles	Investigate the effect of germination on noodle quality	↑ GABA	[7]
			↑ Phenolic acids	
			↓ Resistant starch	
			↓ Cooking time	
			↓ Cooking losses	
			↓ Bitterness	
	Sourdough bread with GBR and *Latilactobacillus sakei*	Evaluate technological suitability of GBR in breadmaking	↑ Phenolic compounds	[9]
			↑ Antioxidant activity	
	WR‐ and GBR‐based snacks	Evaluate nutritional profile of GBR snacks	↑ Digestible starch	[89]
			↓ Undigestible starch	
	Ice cream supplemented with GBR flour	Evaluate GBR flour as a prebiotic ingredient for probiotic ice cream	↑ Sensory properties	[4]
			↑ Probiotic viability during storage	

Dietary supplements	Thai GBR beverage powder extract	Investigate bioactive composition and antitumor activity	Inhibition of tumor cell growth, cell cycle arrest, and reduced migration in cholangiocarcinoma cells	[90]
	Synbiotic formulation based on GBR and probiotics	Evaluate functional properties of a GBR‐based synbiotic	↑ Antioxidant	[91]
			↑ Anti‐inflammatory	
			↑ Glycogen release	
			↑ Cell proliferation; support of probiotic growth and functionality	

Other applications	GBR liquid crystal cosmetic cream	Develop and characterize a GBR‐based moisturizing cosmetic cream	Good stability, moisturizing effect, and suitability for commercial cosmetic applications	[92]
	Brown rice in animal feed	Evaluate rice‐based ingredients as feed supplements	No impairment of pig growth or meat quality	[93]
	Brown rice in aquaculture feed	Investigate rice as an alternative carbohydrate source in aquaculture	Potential reduction in feed costs and improved protein efficiency in fish	[79]

↑ indicates an increase or improvement; ↓ indicates a decrease or reduction.

## 6. Use of GBR for the Formulation of Dietary Supplements

Recently, GBR has received increasing scientific interest as a natural ingredient in the formulation of nutraceuticals and dietary supplements (Table 2). However, scientific evidence in this field remains preliminary since most studies are based on in vitro models and data on both efficacy and stability of GBR‐based supplements at a larger scale are still scarce. In this context, Dangmanee [94] investigated the prebiotic properties of different concentrations of germinated *Sang Yod* BR (1%, 3%, and 5%), reporting that the concentration of 3% was the best in supporting, in vitro, the growth of *Lactiplantibacillus pentosus* GP6. Dokduang et al. [90] investigated the bioactive compound profile of water extracts from powdered germinated Thai rice and assessed its in vitro antitumor properties on cholangiocarcinoma cell lines. The results revealed inhibition of tumor cell growth, cell cycle arrest, and suppression of cell migration, representing preliminary indications of potential antiproliferative activity. Recently, Bosco et al. [91] developed a synbiotic formulation based on GBR and probiotics, investigating its antioxidant, anti‐inflammatory, glycogen release, and cell proliferation properties as well as the ability of GBR to support the growth and functional features of four selected probiotics. Based on preliminary in vitro evidence, the authors suggested that the synbiotic formulation may represent a promising phytotherapeutic strategy in promoting vaginal health and mitigating menopause‐associated dysbiosis, although clinical validation is needed to support any therapeutic claim.

## 7. Other Applications and Future Perspectives

Beyond its established relevance in food and nutraceutical applications, the cosmetic sector may represent a promising area of application for GBR. To date, BR is widely used in formulations for skin improvement [95], due to its content in phenolic compounds and antioxidant activity. To our knowledge, GBR suitability for cosmetic applications is still limited to the study of Jarupinthusophon et al. [92], in which the authors developed an innovative cosmetic cream based on liquid crystals containing GBR or biological cosmetic active ingredients (Table 2). The authors studied its efficacy on hydrating and moisturizing effects; identified the active ingredient content, such as GABA and phenolic content; and evaluated the physical properties and stability of the product. The work suggested that liquid crystal creams containing GBR extract may have potential for use in commercial products, although these findings are based on a single early‐stage formulation study.

In parallel with its emerging potential in cosmetics, rice‐based ingredients have also been investigated in animal feed as a promising feed ingredient or dietary supplement due to the high level of amylopectin, crude protein, calcium, and phosphorus, as well as amino acids [96]. Some studies demonstrated that the use of BR does not compromise the growth or meat quality of pigs (Table 2) [93]. Moreover, BR can also be used in aquaculture as an alternative source of carbohydrates to reduce feed costs and improve protein efficiency in fish (Table 2) [79]. However, although the use of BR in animal feed has been widely explored, studies using GBR as a substitute or supplement in animal diets are limited to date.

## 8. Conclusions and Final Remarks

Cereals or cereal‐derived ingredients, like GBR, have long been staple foods worldwide, and only in recent years has scientific attention shifted toward their health‐promoting potential and the role in disease prevention and well‐being. In particular, the rich nutritional profile of GBR, including DF, polyphenols, minerals, vitamins, inositol, GABA, and *γ*‐oryzanol, contributes to a wide range of beneficial physiological effects, such as antioxidant and anti‐inflammatory activities, antidiabetic, lipid‐lowering, and neuroprotective properties. Beyond its nutritional value, GBR represents an environmentally friendly alternative, promoting a decrease in potentially harmful substances, such as heavy metals and antinutritional factors. Moreover, the prebiotic substances of GBR, which are indigestible in the human colon, may serve as substrates for beneficial gut microorganisms, including lactic acid bacteria, improving the composition of the intestinal microbiota, whose balance has been associated with general well‐being. Although mainly used in functional food formulations, such as beverages, breads, noodles, snacks, and ice cream, GBR may meet different applications beyond food, including nutraceutical supplements, animal feed, and even emerging use in the cosmetics field. However, well‐designed human intervention trials and in‐depth studies are required to reveal the physiological relevance of GBR bioactive compounds and applicability in innovative nutraceuticals tailored to humans’ well‐being.

## Author Contributions

**Georgiana Bosco:** writing – original draft, investigation, data curation. **Valentina Taverniti:** writing – review and editing, visualization, validation, supervision. **Alessandra Pino:** writing – original draft, writing – review and editing, visualization, validation. **Cinzia Caggia:** writing – review and editing, visualization, validation. **Katarzyna Stadnicka:** writing – review and editing, visualization, validation. **Cinzia L. Randazzo:** writing – review and editing, conceptualization, project administration, supervision.

## Funding

This research did not receive any specific grant from funding agencies in the public, commercial, or not‐for‐profit sectors. Open access publishing facilitated by Universita degli Studi di Catania, as part of the Wiley ‐ CRUI‐CARE agreement.

## Ethics Statement

The authors have nothing to report.

## Consent

The authors have nothing to report.

## Conflicts of Interest

The authors declare no conflicts of interest.

## Data Availability

Data sharing is not applicable to this article as no datasets were generated or analyzed during the current study.
